# The Role of Hippocampal Estradiol Receptor-*α* in a Perimenopausal Affective Disorders-Like Rat Model and Attenuating of Anxiety by Electroacupuncture

**DOI:** 10.1155/2016/4958312

**Published:** 2016-12-01

**Authors:** Xun Wang, Yongheng Huang, Shiwen Yuan, Amin Tamadon, Shulan Ma, Yi Feng

**Affiliations:** ^1^Department of Integrative Medicine and Neurobiology, State Key Laboratory of Medical Neurobiology, Shanghai Medical College, Institute of Acupuncture Research (WHO Collaborating Center for Traditional Medicine), Institutes of Brain Science, Brain Science Collaborative Innovation Center, Fudan University, Shanghai 200032, China; ^2^2008 Clinical Medicine, Shanghai Medical College, Fudan University, Shanghai 200032, China

## Abstract

Hormone replacement therapy is the principal treatment for perimenopausal affective disorders which can cause severe side effects. The present study compared the effects of electroacupuncture (EA) and estradiol treatment on perimenopausal affective disorders at the behavioral and cellular levels. In this randomized experimental* in vivo* study, adult female rats were divided into intact, ovariectomy, chronic unpredictable stress (CUS), and ovariectomy and CUS combination groups. After week 6, all groups were subdivided to three subgroups of control, EA, and estradiol treatment. The behavioral parameters in the open field and the elevated plus maze tests were assessed before and after treatments. Alterations of serum steroid hormones and changes of estradiol receptor-*α* (ER-*α*) immunofluorescence neurons in the hippocampus sections were evaluated. EA treatment caused more antianxiety effects than estradiol treatment in CUS group (*P* < 0.05). Notably, estradiol and EA treatments had better significant behavioral effects when the models were not estrogen-deficient. Importantly, within each group, compared to the control group, the numbers of ER-*α*-positive neurons were significantly larger in EA subgroups. Therefore, EA had antianxiety effects on perimenopausal affective disorders caused by CUS but not by estrogen deficiency and upregulation of hippocampus ER-*α* neurons may contribute to its mechanism of action.

## 1. Introduction

Menopause is associated with irregular menstrual cycles, widely fluctuating hormone levels, and hypothalamus-pituitary-ovary axis disorder [1]. Perimenopausal syndrome may appear up to 2 to 8 years before menopause and subside 1 year after final menstruation [2]. Symptoms of the syndrome include the physiological symptoms of hot flashes, sweating, insomnia, and somatic pain and the psychological symptoms of depression and anxiety [3]. Several studies suggested a role of sex steroid hormones in the onset of depressions in the menopause transition which are reviewed by Schmidt and Rubinow [4]. Currently, hormone replacement therapy (HRT) is the principal treatment for perimenopausal symptoms, but HRT can cause severe side effects, such as breast cancer [5]. Additionally, under certain circumstances, environmental exposure to exogenous estrogens may play a role as an endocrine disruptor and adversely affect reproductive outcome [6].

Acupuncture, the long-tested traditional Chinese medical procedure, may be an alternative treatment for perimenopausal symptoms; it causes fewer side effects than conventional therapy and is becoming more common [5, 7]. It also has shown beneficial effects in the treatment of psychological disorders [5, 8]. The mechanisms of the effects of acupuncture on perimenopausal depression are not clear, but it has been shown that electroacupuncture (EA) is able to increase circulating estrogen concentrations [9]. Recent studies showed that acupuncture treated menopausal syndrome and improved memory subsequently [10, 11]. The acupuncture also increased plasma and brain estrogen levels [11–13], also shown to improve memory and learning in rat [11]. In addition, the hippocampus is one of the most important regions of the central nervous system which controls emotion and memory, and the hippocampus is likely a central target for the effects of estradiol to reduce anxiety and depression [14]. The hippocampus is an important component of the limbic system and regulator of the hypothalamic-pituitary-adrenal (HPA) axis responses that its manipulation by steroid hormones had effect on anxiety and depression [15]. Furthermore, the positive effect of EA on anxiety behaviors via alterations of some molecular pathways in hippocampus in other rat models related to depression, stress, or pain has been shown [16–18].

It has been shown that, at the perimenopausal stage, the numbers of neurons with estrogen receptors (ERs) significantly changed [19]. The most commonly occurring isoforms of the ERs are ER-*α* and ER-*β*. The majority of the effects that estradiol exerts upon multiple brain circuits are mediated by ER-*α* [20]. Although in the hippocampal cell culture, activation of either ER-*α* or ER-*β* resulted in neuroprotection [21], but their distribution in hippocampal neurons is not completely overlapped. ER-*α* is expressed in nuclear and extranuclear sites of principal and inhibitory neurons [22], while extranuclear ER-*β* is expressed mostly in principal cells [23]. ER-*α* but not ER-*β* showed an increasing pattern in the hippocampal cells during postpartum period in rats which shows the role of ER-*α* in onset of anxiety-like and depression-like behaviors [24]. ER-*β* modulates ER-*α*'s ability to elicit receptive and proceptive sexual behavior [25]. Furthermore, studies on ER-knockout mice or selective ER modulators also showed that the ER-mediated effects of estradiol on anxiety and depressive behaviors may require ER-*β* [15, 26].

Since hippocampal ER-*α* may participate in the treatment effects of acupuncture on perimenopausal depression, EA effects on the perimenopausal affective disorders which were induced by estradiol deficiency and/or stress and additionally the ER-*α* expression on the neurons in the cortex hippocampus as one of the possible pathways of EA therapeutic effects were evaluated.

## 2. Materials and Methods

### 2.1. Animals and Ethics

Sixty-one adult female Sprague-Dawley rats (8 weeks old) were purchased from Shanghai Laboratory Animal Center (SLAC) (Shanghai, China). The rats were housed as three to four per cage under controlled conditions (21-22°C, 55–65% humidity, and 12 h light/12 h dark cycle). All of the animals had free access to tap water and were fed* ad libitum* with standard laboratory chow (SLAC, Shanghai, China) that did not contain alfalfa or soybean meal, thus minimizing the occurrence of natural phytoestrogens. Body weights were measured weekly with an electronic weight balance. The weights were recorded once the digits displayed were stable. This study was approved by the Animal Ethics Committee of Shanghai Medical College, Fudan University.

### 2.2. Experimental Design and Study Procedures

**Figure 1:**
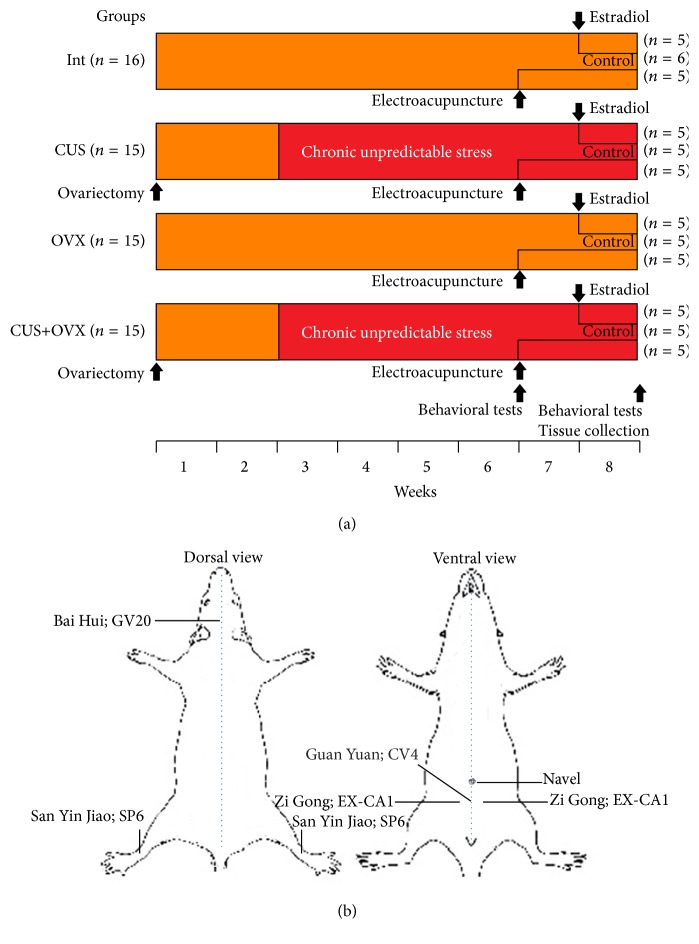
(a) Schematic diagram of the experimental design including the behavioral tests, treatments, and tissue collection for evaluation of the role of hippocampal estradiol receptor-*α* on perimenopausal affective disorders and their treatment by electroacupuncture and estradiol in rats. INT, intact; CUS, chronic unpredictable stress; OVX, ovariectomy; OVX + CUS, ovariectomy and chronic unpredictable stress. (b) Schematic drawing showing the location of the acupuncture points used in the present study. Bai Hui (GV20) is located at the right midpoint of the parietal bone. San Yin Jiao (SP6) is located on the medial lower leg, above the prominence of the medial malleolus. Guan Yuan (CV4) is located on the midpoint from umbilicus to symphysis pubis. Zi Gong (EX-CA1) is located lateral to the ventral midline and superior to the upper border of the pubic symphysis.

The rats were randomly divided into four groups (Figure 1(a)): intact (INT, *n* = 16), chronic unpredictable stress (CUS, *n* = 15), ovariectomy (OVX, *n* = 15), and ovariectomy and chronic unpredictable stress (OVX + CUS, *n* = 15). After three days of acclimatization, the rats in the OVX and OVX + CUS groups were ovariectomized, and vaginal smears were taken daily for the following two weeks to assess the success of the ovariectomy. From week 3 to week 8 after ovariectomy, chronic unpredictable stress was applied to the CUS and OVX + CUS groups. During weeks 7 and 8, the four initial groups were divided into three treatment subgroups: EA (*n* = 5), estradiol (*n* = 5), and control (*n* = 5; for INT group, *n* = 6). EA groups were treated with EA from week 7. Estradiol groups were treated with oral-intake estradiol from week 8. Finally, the controls did not receive any treatment. There was no overlap of treatments within each subgroup.

The behavioral tests were performed at the start of week 7 (before EA treatment) and at the end of week 8 (2 weeks after EA treatment and before sampling). Body weight was recorded weekly during the experiment.

### 2.3. Ovariectomy and Sham Operation

The ovaries of the rats in the OVX and OVX + CUS groups were removed under isoflurane anesthesia. Bilateral incisions were made in the dorsolateral skin and muscle wall. The ovaries and fallopian tubes were ligatured and excised. The muscle wall and then skin were sutured. The OVX rats recovered for 1 week with daily postoperative monitoring. Buprenorphine (0.05 mg/kg, SC) was injected as a pain reliever immediately after surgery and repeated every 12 hours for 3 days. Successful operations were confirmed when the vaginal smear tests showed no mature exfoliated epithelial cells for 10 continuous days after the procedure according to the method of Walf and Frye [27]. The rats in intact and CUS groups were also submitted to the same surgery procedure and some adipose tissues around their ovaries were removed.

### 2.4. Chronic Unpredictable Stress

The rats were isolated in single cages and began receiving chronic unpredictable stress procedures during weeks 3 to 8. Seven different procedures were applied within six weeks. Each rat encountered one or two stresses per day, and the unexpectedness of the stress was guaranteed by the random onset of the procedures, which included broiling (40°C, 15 min), humidified cushion (12 h), cold water swimming (4°C, 5 min), noise (6 h), quaking (30 min), deprivation of food and water (12 h), and reversal of light and dark cycles according to the method that was explained by Chen et al. [28].

### 2.5. Behavioral Tests

The same rats were used for all behavioral tests, which were performed in the following order: open field and elevated plus maze tests. All of the experiments were performed under white light during the light phase of the light/dark cycle, and behavior was recorded with a video camera (Sony, HD110) for further analysis. The rats in the EA and the control groups were tested during the estrous phase of their cycle. The behavioral observations were carried out under blind conditions.

#### 2.5.1. Open Field Test

The open field apparatus (60 cm × 60 cm area and 60 cm high wall) was divided into 16 squares by black lines. An area consisting of the inner four squares (30 cm × 30 cm), which was not attached to the wall of the apparatus, was designated as the central part, whereas the area surrounding the center was designated as the peripheral part. The illumination at floor level was 60 lux. At the beginning of the test, a rat was removed from the home cage and gently placed in a corner square with her head facing the corner in the open field. Movement of the animal in the arena during the 10-minute testing session was recorded. Behavioral variables which were measured in the open field test include the following:

- Line crossings: frequency with which the rat crossed a grid line with all four paws. A high frequency of this behavior indicates increased locomotion and exploration and/or a lower level of anxiety [29]
- Grooming and grooming time: frequency and duration with which the rats scratched, licked, or bit their coat, whiskers, feet, or tail. A high frequency and time of this behavior indicate higher level of anxiety [30]
- Central part entries and duration: frequency and duration with which rat with all four paws entered into the central square. A high frequency and time of this behavior indicate lower level of anxiety [31]
- Peripheral part entries: frequency with which rat spent time in the periphery of the arena. A high frequency of this behavior indicates higher level of anxiety [32]
- Rearing: frequency with which the rat stands vertically on two hind legs. A high frequency of this behavior indicates increased locomotion and exploration and/or a lower level of anxiety [29]

After 10 min, the animal was removed from the apparatus and returned to the home cage. The open field arena was cleaned to prevent olfactory cues from affecting the behavior of subsequently tested rats. The procedure was performed according to Carobrez and Bertoglio [33].

#### 2.5.2. Elevated Plus Maze Test

The movements of the rats in an elevated cross bar with two open arms (50 cm long × 10 cm wide) and two closed arms (50 cm long × 10 cm wide) were recorded. The two closed arms were enclosed by 38 cm high black Plexiglas walls, whereas the two open arms had transparent plastic ledges (0.3 cm) to prevent the rats from falling. The wall of closed arm was made from dark polyvinylchloride and was placed 73 cm above the floor in a silent and dimly illuminated room (3 m × 4 m; light intensity: open arms 100 lux, closed arms 60 lux). A rat was placed directly at the junction of the open and closed arms and was allowed to explore undisturbed for 10 min while being videotaped:

- Close arm entries and duration: frequency and duration with which rat with all four paws entered into the close arm. A high frequency and time of this behavior indicate higher level of anxiety [34]
- Total arm entries: frequency with which rat entered into the arms. A high frequency of this behavior indicates higher level of anxiety [34]
- Open arm entries: frequency with which rat with all four paws entered into the close arm. A high frequency of this behavior indicates lower level of anxiety [34]
- Stretched-attend postures: the number of stretched-attend postures in the open arms and the closed arms were assessed. A high frequency of this behavior indicates higher level of anxiety [35]
- Rearing: frequency with which the rat stands vertically on two hind legs. A high frequency of this behavior indicates lower level of anxiety [36]
- Grooming: frequency with which the rats scratched, licked, or bit their coat, whiskers, feet, or tail. A high frequency and time of this behavior indicate higher level of anxiety [37].

The maze was carefully cleaned with water between each separate trial. The procedure was done based on the methods of Walf and Frye [38] and Albani et al. [39].

### 2.6. Electroacupuncture Treatment

Under quiet circumstances, the rats were situated in the EA equipment, in which the heads and the limbs of the rats were free. The selected acupuncture points were “Bai Hui” (GV20), “San Yin Jiao” (SP6), “Guan Yuan” (CV4), and “Zi Gong” (EX-CA1) (Figure 1(b)). A 2 Hz interflow electrical stimulation was applied to the needles in “San Yin Jiao” (SP6) and “Zi Gong” (EX-CA1). The electrical intensity that caused a slight rhythmic tremor of the feet was considered suitable. The treatment lasted for 30 min and was performed for 10 days based on the previous reports [40–42]. The estradiol and control subgroups were restrained in the same equipment for 30 min each day for 10 days such as EA subgroups.

### 2.7. Estradiol Treatment and Vehicle Administration

Estradiol tablets (Progynova®, Bayer Schering Pharma, production number: 201A) were ground and then dissolved in corn oil, and 0.2 mL of the solution, containing 0.02 mg of estradiol valerate, was administered daily to the rats by gavage [43]. The EA and control subgroups were given corn oil by gavage. Treatment lasted for 5 days and ended with EA treatment.

### 2.8. Tissue Collection

The female rats were weighted and then euthanized with chloral hydrate at the end of week 8. Then tissues including uteruses, ovaries, and adrenal glands were collected and weighted. Arterial blood was collected from aorta abdominalis and stored overnight in 4°C refrigerator and then centrifuged to obtain supernatants for circulating hormones estradiol, testosterone, and cortisol. Right after the blood collection, perfusion was done with 300 mL physiological saline and 200 mL paraformaldehyde to collect the brain tissue. Coronal sections were made for immunofluorescence staining.

### 2.9. Preparation of Brain Sections

For immunofluorescence staining, the rats were deeply anesthetized with chloral hydrate and then perfused via the left cardiac ventricle with 300 mL of 4°C 0.9% sodium chloride and 200 mL of 4% paraformaldehyde to quickly fix the tissue. The rats' brains were dissected and postfixed in 0.1 M PBS containing 20% sucrose for 24 h at 4°C and then in 0.1 M PBS containing 30% sucrose for 24 h at 4°C, followed by 4% paraformaldehyde containing 30% sucrose at 4°C until the brain sank, indicating dehydration. Serial frozen coronal sections (35 *μ*m) were collected (Leica CM1900, Germany) and were stored in tissue culture wells containing 30% sucrose and 30% ethylene glycol in 0.1 M PBS (pH 7.4) at −20°C.

### 2.10. Brain Immunofluorescence Microscopy and Analysis

Free-floating sections were washed for 3 × 5 min in Tris buffered saline (TBS; 20 mM Tris, 0.9% NaCl, pH 7.4), followed by incubation in 0.5% Triton X-100 for 15 min at room temperature and 4% normal horse serum for 2 h at 37°C. The sections were then incubated with the primary antibody (rabbit anti-ER-*α*: 1 : 50, Santa Cruz Biotechnologies, Santa Cruz, CA) for 1 h at 37°C and then overnight at 4°C. After rinsing, the sections were incubated with an appropriate fluorescent secondary antibody (Alexa Fluor 594 donkey anti-rabbit IgG H + L, 1 : 125, Invitrogen) for 1 h at 37°C. After six rinses, the sections were mounted and sealed. The sections were imaged on a Leica DM LB2 microscope (Germany) under an ultraviolet light source and were photographed using Spot Advanced software (version 4.6, Diagnostic Instruments, Inc.). The brain sections at the Interaural line 5.70 mm Bregma −3.0 mm were observed according to Paxinos and Watson [44]. The number of ER-*α*-positive neurons was counted in 7 to 8 sections from each animal. All of the immunofluorescent assays included a comparison without primary antibody. To ensure comparability of the immunostaining, brain sections with different experimental groups or the same modeling procedure with different treatments were processed concurrently. Care was taken to ensure that the area of the regions selected for comparison did not differ between sections and subjects.

### 2.11. Assessment of Circulating Hormones

Using radioimmunoassay kits, the serum levels of estradiol (I^125^-estradiol RIA Kit; 201105 1106), testosterone (I^125^-testosterone RIA Kit; 201105 1003), and cortisol (I^125^-cortisol RIA Kit; 201105 1007) were determined using radioimmunoassay kits and were examined by the Beijing Fu Rui Biology Company, China. The RIA assay results showed the following: for estradiol, NSB < 5%, sensitivity < 5 pg/mL, inter-CV < 10%, and intra-CV < 15.2%; for testosterone, NSB < 5%, sensitivity < 0.1 ng/mL, inter-CV < 8%, and intra-CV < 15%; and for cortisol, NSB < 5%, sensitivity < 1 ng/mL, inter-CV < 7.5%, and intra-CV < 9.5%.

### 2.12. Statistical Analysis

The data are expressed as the means ± standard error of means. Comparisons between the data were performed using two-way ANOVA and LSD* post hoc* test (SPSS, version 22; Chicago, IL). A value of *P* < 0.05 was set as the limit of statistical significance. After evaluations of behavioral tests' parameters in each group according to changes of means in the posttreatment subgroups in comparison with the controls before treatment, a scoring system was designed to evaluate the effect of treatments on behavior parameters.

## 3. Results

### 3.1. Body and Tissue Weights

Ovariectomy increased the body weights compared to the INT and CUS groups (Table 1). For the rats whose ovaries were intact (INT and CUS groups), estradiol treatment decreased the weight of their ovaries (*P* < 0.05, Table 1). EA treatment increased the mean weight of adrenal glands and mean body weight in OVX, CUS, and OVX + CUS groups; increase in the weight of adrenal glands was significant in OVX + CUS group in comparison with concurrent control subgroups (*P* < 0.05, Table 1).

### 3.2. Behavioral Alterations of OVX Group

**Figure 2:**
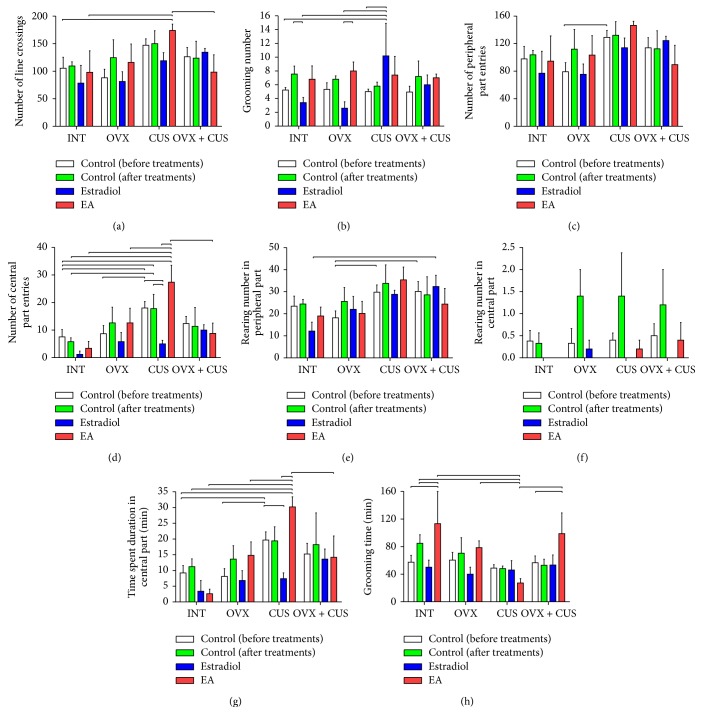
Comparisons between means and standard errors of behavioral changes in open field test to evaluate the effects of electroacupuncture and estradiol on perimenopausal affective disorders in rats. INT, intact; OVX, ovariectomy; CUS, chronic unpredictable stress; EA, electroacupuncture. Significant differences between related groups are shown by lines (*P* < 0.05).

**Figure 3:**
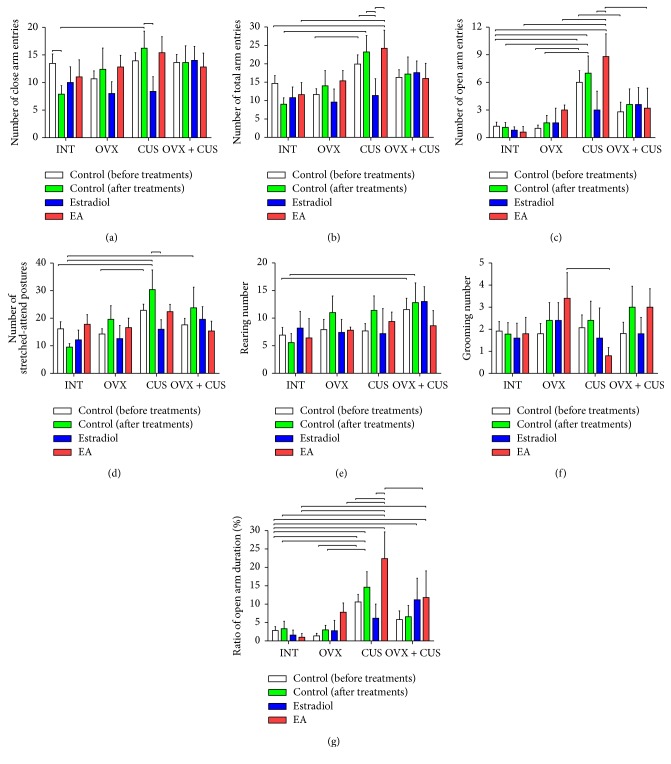
Comparisons between means and standard errors of behavioral changes in elevated plus maze to evaluate the effects of electroacupuncture and estradiol on perimenopausal affective disorders in rats. INT, intact; OVX, ovariectomy; CUS, chronic unpredictable stress; EA, electroacupuncture. Significant differences between related groups are shown by lines (*P* < 0.05).

The alterations of evaluated behavior parameters in open field arena and elevated plus maze are shown in Figures 2 and 3. Overall antianxiety effects of estradiol and EA treatments and anxiety changes of control group after treatment in three groups of OVX, CUS, and OVX + CUS which were compared with control groups before treatment are summarized in Table 2. The OVX control group after treatment did not show an anxiety behavioral outcome in comparison with control before treatment totally (Figures 2 and 3 and Table 2). On the other hand, EA treatment (10/15 parameters) increased means of anxiety behaviors in the OVX rats in comparison with estradiol treatment (6/15 parameters). It should be emphasized that these observations were not significantly different between groups and are reported just based on the means' alterations.

### 3.3. Behavioral Alterations of CUS Group

The behavioral outcome of CUS in the control rats after treatment was anxiety, which was characterized by comparison with before-treatment control group (Figures 2 and 3 and Table 2). Furthermore, EA treatment decreased anxiety behaviors in comparison with estradiol (Table 2). For instance, time and frequency of entrance into central parts in open field test and frequency and ratio of open arm entries in elevated plus maze as indicators of anxiety increased (*P* < 0.05, Figures 2(d), 2(g), 3(c), and 3(g)). Otherwise, estradiol treatment reduced the time spent and frequency in central part of open field arena and frequency and ratio of open arm entries in elevated plus maze in CUS group (*P* < 0.05, Figures 2(d), 2(g), 3(c), and 3(g)). Regarding the increasing in grooming behavior as an index of anxiety in CUS group, estradiol increased grooming frequency and EA decreased grooming time (*P* < 0.05, Figures 2(b), 2(h), and 3(f)). In contrast to the mentioned parameters, number of line crossings in open field arena increased by EA treatment in CUS group which shows increase of anxiety (*P* < 0.05, Figure 2(a)). Furthermore, the stretched-attend postures, number of close arm entries, and total arm entries as other indices of increase in anxiety in elevated plus maze reduced by estradiol treatments (*P* < 0.05, Figure 3(d)).

### 3.4. Behavioral Alterations of OVX + CUS Group

Combination of CUS and OVX in most findings of behavioral tests in control groups presented the predominance of anxiety behaviors after comparison with control group before treatment (Figures 2 and 3 and Table 2). On the other hand, EA treatment (9/15 parameters) increased means of anxiety behaviors in the OVX + CUS rats in comparison with estradiol treatment (7/15 parameters; Table 2). In the OVX + CUS group, analyzing of open field test results after treatments showed that EA increased time of grooming in comparison with before-treatment control group (*P* < 0.05, Figure 2(h)). In addition, the rats which received estradiol and EA treatment in the OVX + CUS group spent more time in open arm of elevated plus maze (*P* < 0.05, Figure 3(g)).

### 3.5. Serum Steroid Hormones

**Figure 4:**
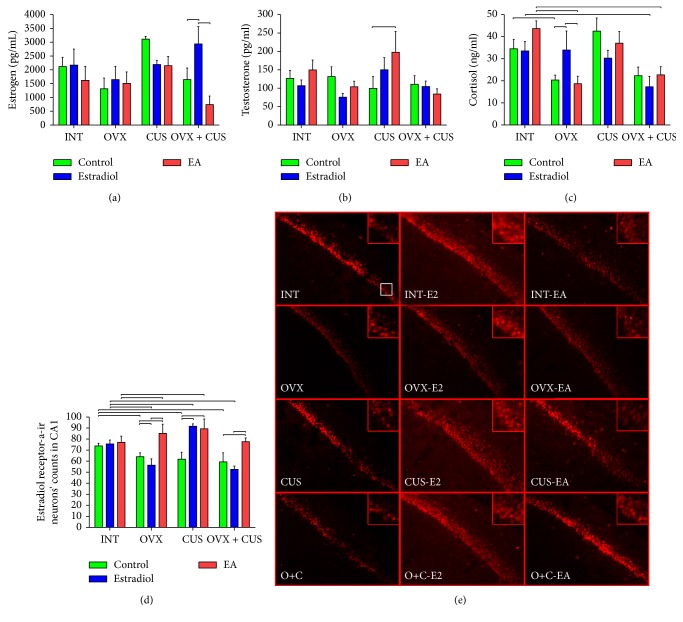
Comparisons between mean and standard error of serum steroid hormones (a–c) and estradiol receptor-*α* immunoreactive neurons' counts in CA1 hippocampus (d) to evaluate effect of electroacupuncture and estradiol on perimenopausal affective disorders in rats. Representative immunofluorescent photos indicating estradiol receptor-*α* immunoreactive neurons in CA1 hippocampus for evaluation of the effect of electroacupuncture and estradiol on perimenopausal affective disorders in rats (e). Significant differences between groups are shown by lines (*P* < 0.05). INT, intact group; CUS, chronic unpredictable stress group; OVX, ovariectomy group; O + C, ovariectomy and chronic unpredictable stress group; EA, electroacupuncture; E_2_, estradiol.

Evaluation of serum estradiol tests showed that estradiol treatment could increase estrogen concentration just in the OVX + CUS group (*P* < 0.05, Figure 4(a)). On the other hand, testosterone concentrations increased by EA and estradiol treatments in CUS group (*P* < 0.05, Figure 4(b)). In addition, ovariectomy reduced the serum cortisol concentrations in OVX group, but estradiol replacement prevented its decrease (*P* < 0.05, Figure 4(c)). On the other hand, combination of CUS with ovariectomy in OVX + CUS group did not permit increase of cortisol concentrations in estradiol replacement subgroup. However, there were trends in decrease of estrogen in CUS + OVX group and EA logically could not increase the serum estradiol concentrations such as estradiol replacement, but EA could induce increase of ER-*α* in neurons of CA1 hippocampus (Figure 4(d)).

### 3.6. Immunofluorescence

As shown in Figure 4(e), the CA1 region of the hippocampus manifests a large number of ER-*α*-positive neurons, which were oval, bipolar, or triangular in shape. Within each group, compared with the control group, the thicknesses of the CA1 region of the hippocampus after EA or estradiol treatments were larger with increased number and volume of ER-*α*-positive neurons (Figure 4(e)). All of the EA treatment groups showed increased cell layers and density. For the estradiol treatment subgroups, the OVX and the OVX + CUS groups in comparison with the CUS group showed fewer immunoreactive neurons. In the OVX and the OVX + CUS groups, ER-*α* was present in the cytoplasm of neurons, while the other two groups demonstrated a distribution of red fluorescence, especially for the EA and estradiol subgroups of INT and CUS groups (Figure 4(e)).

## 4. Discussion

As for the results after treatments, EA treatment eminently ameliorated anxiety-like behaviors in the CUS group, while estradiol treatment showed no antianxiety effects under the same conditions. On the other hand, comparing the effects of estrogen treatment and EA on behavioral indices including number and the spent duration in central part of open field arena and frequency and ratio of open arm entries in the elevated plus maze, CUS subgroups indicated better effect of EA than estradiol treatment. These results concur with the opinion that EA can produce HRT-like reversal on some perimenopausal anxiety-like behavior but with much milder effects [5].

Women with perimenopausal symptoms on acupuncture therapy showed decreased levels of follicle stimulating hormone and luteinizing hormone in the serum but increased levels of estradiol which may led to reduction of anxiety and improvement of perimenopausal symptoms [45, 46]. As the estrogen concentrations could not be increased by EA in the OVX and OVX + CUS animals, antianxiety effects were not observed in those models. In contrast, in CUS model, similarity of estrogen concentrations in EA and estradiol treatment groups and also increase of antianxiety effects of EA in this group demonstrate the possible mechanism of EA in reducing perimenopausal affective disorders by increasing estrogen concentrations. Notably, EA treatments had a better effect when the models were not estrogen-deficient. The most popular theory of a possible EA treatment mechanism is that it motivates the body's own potential physiological functioning to offset certain impairments [47]. Therefore, it is not rash to propose that the EA treatment's effect was not as vulnerable as the estrogen treatment to the influences of basic hormone levels.

Regarding the animal modeling, CUS and CUS + OVX both induced anxiety-like symptoms in the rats. On one hand, during the CUS procedure, the rats were subjected to external physical stresses, which put them into a nervous state (which can be inferred from the anxiety indices increase from the behavioral tests). Additionally, some items of the CUS, to some extent, physically exercised the rats and thus increased their mobility, which might influence the results of the behavioral tests.

On the other hand, the major effects of OVX on the rats were the drop in hormone levels, which is considered as physiological stresses. Moreover, the reduction of estrogen in the OVX rats caused their body weights to increase and this side effect may lower the ability of OVX rats to move. Comprehensively speaking, the reason why no significant differences were found in the OVX + CUS group may be due to the “behaviorally counterbalanced” effect, that is, enhanced and decreased locomotor activities of the CUS and the OVX on the rats.

Furthermore, decrease in ER-*α*-positive neuron in OVX, CUS, and OVX + CUS groups indicated the role of this receptor in premenopausal affective disorders. Consistent with our findings, ER-*α* is reported to participate in generating anxiety-like and depression-like behaviors [48]. The immunofluorescence results showed that OVX and CUS are capable of causing a loss of cells and a volume decrease in the limbic system, as observed in the hippocampus [19, 49]. The increase in the ER-*α*-positive neuron numbers in the CA1 region of the hippocampus in the EA groups indicated the EA treatments' mechanism of function in this disorder. It is shown that EA can enhance the growth or mitosis of functional rat hippocampal neurons or prevent their apoptosis [50–52]. EA treatment had better inducing effects on OVX and OVX + CUS groups than estradiol in increase of ER-*α*. Consistent with our findings, EA increased ER expression in ovariectomized animals [52, 53]. Previous studies have shown that EA of “San Yin Jiao” (SP6) and “Guan Yuan” (CV4) upregulated estrogens in rats' uterus and ovary by affecting the HPA axis [40, 41, 54]. Therefore, acupuncture may have a therapeutic impact on ER-associated dysfunctions.

In addition, our findings showed that the clustering of fluorescent particles reflects the activation of the ER-*α* receptors. ER-*β*, another estrogen receptor, was expressed to a lesser degree in the hippocampus and its expression was not significantly different between the groups and subgroups (data not shown). For the OVX rats, the neuron effect of estradiol was not as significant as it was for intact rats. The literature reported that estradiol treatment has a “therapy window”; the effect of HRT after menopause declines as the time interval between the onset of menopause and the treatment becomes longer [55–57]. This might explain the discord between the behavioral and cellular results of EA and estradiol treatment effects on OVX rats.

Furthermore, the data from Figures 2 and 3 seems to rule out the anxiety factors from affective disorders of the OVX rats; we know that the indices of anxiety symptoms are largely influenced by the mobility of the rats, as explained above, and thus it would be impetuous to draw the conclusion that OVX would not induce total anxiety-like behaviors. On the other hand, indispensable discussion regards the general motor ability of the model rats. A study by Walf and Frye [14] parsed out the motor ability factors that may bias the evaluation of affective disorders in behavioral tests. That is to say, estrogen treatment does not ameliorate affective disorders by enhancing general motor ability. If the open arm entries in the elevated plus maze are considered as an index of the motor ability of rats [58], then, as seen from Figure 3(c), CUS significantly increased the mobility of the model rats and this might be an important factor affecting the evaluation of the behavioral tests.

Regarding body and tissue weights, six-week CUS decreased the body weight and did not have effect on adrenal gland weight. Consistent with our findings, decreasing effect of CUS in body weight has been reported in both mice and rats and this weight loss is related to stress [59, 60]. Otherwise, increasing in adrenal gland after 8-week stress in mice and also increase in ratio of adrenal weight to body weight in rats have been reported [59, 60] which were in contrast with our findings that no significant alterations were observed in the adrenal weight or in its ratio to body weight (data are not shown) considering the reduction of body weight. This phenomenon may be influenced by increasing the duration of CUS.

On the other hand, the EA treatment in all groups increased the weight of adrenal glands and decreased the body weight. The same effects of EA have been previously reported [61, 62]. In contrast with previous report [61], cortisol concentrations did not increase significantly in EA groups. This finding can be explained by the selected acupoints which could reduce anxiety directly via brain.

In conclusion, although in the OVX + CUS group, the estradiol replacement increased the estradiol concentrations and induced anxiety effects but EA treatment by increasing of ER-*α* in CA1 hippocampus compensated the lower concentrations of estradiol and also induced nonanxiety effects in the rats. Consistent with our study, comparison of the effects of estrogen replacement and acupuncture in menopausal symptoms in women showed that EA significantly reduced the hot flushes frequency [63]. The EA treatment's effect is not vulnerable to basic estrogen level influences. Therefore, the EA treatment had antianxiety effects on perimenopausal affective disorders caused by CUS but not by estrogen deficiency and upregulation of hippocampus ER-*α* neurons may contribute to its mechanism of action.

## Figures and Tables

**Table 1:** Comparisons between mean and standard error of body and tissue to evaluate effect of electroacupuncture on perimenopausal affective disorders in rats.

Groups	Body weight (g, week 1)	Subgroups	Weights (g, week 8)			
			Body	Uterus	Ovary	Adrenal
INT	181.4 ± 1.6	Control	287.3 ± 2.4	533.5 ± 33.6	125.4 ± 3.9	72.5 ± 8.8
		Estradiol	261.5 ± 13.4	468.3 ± 43.5	96.8 ± 6.1^*∗*†^	61.4 ± 1.2^*∗*^
		EA	268.6 ± 10.1	441.1 ± 34.9	118.6 ± 4.3^†^	63.0 ± 3.0
OVX	184.9 ± 1.6	Control	334.0 ± 12.8^#^	ND	ND	58.4 ± 3.4^#^
		Estradiol	307.2 ± 11.6	ND	ND	68.3 ± 9.1
		EA	305.1 ± 9.3	ND	ND	65.9 ± 6.4
CUS	182.6 ± 1.4	Control	263.8 ± 1.5^#^	484.7 ± 47.0	107.6 ± 10.0	61.4 ± 6.2
		Estradiol	267.4 ± 4.9	491.4 ± 39.3	86.5 ± 4.1^*∗*^	63.2 ± 7.0
		EA	260.8 ± 3.1	564.5 ± 61.0	92.0 ± 4.6	75.7 ± 3.8
OVX + CUS	181.3 ± 1.7	Control	305.4 ± 7.9	ND	ND	55.2 ± 5.6^#^
		Estradiol	318.0 ± 6.2^#^	ND	ND	78.6 ± 6.0^#*∗*^
		EA	291.2 ± 11.6	ND	ND	80.4 ± 7.6^#*∗*^

INT, intact; OVX, ovariectomy; CUS, chronic unpredictable stress; EA, electroacupuncture; ND, no data.

^*∗*^Significant difference with internal control (*P* < 0.05).

^#^Significant difference with the similar subgroups in intact group (*P* < 0.05).

^†^Significant difference between EA and estradiol treatments in each group (*P* < 0.05).

**Table 2:** Comparisons between two types of therapeutics on attenuating of anxiety and depression behaviors in perimenopausal affective disorders in rats based on various patterns of behavior tests.

Tests and patterns	OVX			CUS			OVX + CUS		
	Control	E_2_	EA	Control	E_2_	EA	Control	E_2_	EA
Open field test									
Line crossings	+	−	−	+	−	+	−	+	−
Grooming	−	+	−	−	−	−	−	−	−
Peripheral part entries	−	+	−	−	+	−	−	+	−
Central part entries	+	−	+	−	−	+	−	−	−
Rearing in peripheral part	+	+	+	+	−	+	−	+	−
Rearing in central part	+	−	−	+	−	−	+	−	−
Duration in central part	+	−	+	−	−	+	+	−	−
Grooming time	−	+	−	=	+	+	+	+	−
Elevated plus maze test									
Close arm entries	−	+	−	−	+	−	=	−	+
Total arm entries	−	+	−	−	+	−	−	−	+
Open arm entries	+	+	+	+	−	+	+	+	+
Stretched-attend postures	−	+	−	−	+	+	−	−	+
Rearing	+	−	−	+	−	+	+	+	−
Grooming	−	−	−	−	+	+	−	−	+
Open arm duration ratio	+	+	+	+	−	+	+	+	+
Overall antianxiety effect	+1	+3	−5	−2	−3	+5	−2	−1	−3

INT, intact; OVX, ovariectomy; CUS, chronic unpredictable stress; Co, control; E_2_, estradiol; EA, electroacupuncture.

^*∗*^Differences of mean of behavior patterns between treatments and pretreatment control in each group: =, no effect; +, antianxiety effect; −, anxiety effect.
